# Comparative Transcriptome Analysis Between a Spontaneous Albino Mutant and Its Sibling Strain of *Cordyceps militaris* in Response to Light Stress

**DOI:** 10.3389/fmicb.2018.01237

**Published:** 2018-06-08

**Authors:** Fen Wang, Qing Liu, Jiaojiao Zhang, Kuanbo Liu, Kuan Li, Guijun Liu, Caihong Dong

**Affiliations:** ^1^State Key Laboratory of Mycology, Institute of Microbiology, Chinese Academy of Sciences, Beijing, China; ^2^College of Life Sciences, University of Chinese Academy of Sciences, Beijing, China; ^3^Beijing Radiation Center, Beijing, China

**Keywords:** albinism, *Cordyceps militaris*, transcriptome, light stress, replication and repair

## Abstract

Albinism has been used for new variety screening in some edible mushrooms and the underlying mechanisms are fascinating. Albino fruiting body of *Cordyceps militaris*, a well-known edible fungus and model organism for Cordyceps, has the potential to be a nutraceutical or functional food due to its high content of metabolites and antioxidant activities. In this study, a spontaneous albino mutant strain (505) of *C. militaris* was obtained. In comparison to its normal sibling strain (498), the albino strain stably remained white in response to light and had significantly decreased conidia and carotenoid production but accumulated more cordycepin. Transcriptome analysis of both strains revealed that all the seven photoreceptors were expressed similarly in response to light. However, many more genes in the albino strain were differentially expressed in response to light than its sibling strain. The significantly enriched pathways in 498L vs. 505L were mainly associated with replication and repair. Some secondary metabolite backbone genes including those encoding DMAT, two NRPS-like proteins, three NPRS, and lanosterol synthase were differentially expressed in the albino when compared with that of the normal strains. Transcriptome and real-time quantitative PCR analyses indicated that some cytochrome P450s and methyltransferases might be related to the phenotypic differences observed between the two strains. This study compared the genome-wide transcriptional responses to light irradiation in a spontaneous albino mutant and its normal sibling strain of an edible fungus, and these findings potentially pave the way for further investigation of the pigment biosynthetic pathway.

## Introduction

*Cordyceps militaris* (L.) Fr., a highly valued edible and medicinal fungus is known to generally parasitize the larvae or pupae of lepidopteran insects. It has been widely used as a food tonic in East Asia as well as a substitute for *Ophiocordyceps sinensis* (syn. *Cordyceps sinensis*) in traditional Chinese medicine. Recent studies have confirmed its biological significance such as its anti-tumor (Liu et al., 2017), anti-inflammatory (Yoon et al., 2015; Chiu et al., 2016), antioxidant, antibacterial, and antifungal activities (Reis et al., 2013). It has been approved as a novel food by the Ministry of Health of the People’s Republic of China.

The fruiting bodies of this fungus have been successfully produced industrially and commercialized. Natural and cultivated fruiting bodies of *C. militaris* appear yellow to orange colored. Carotenoids, including lutein, zeaxanthin and four cordyxanthins, have been isolated from the *C. militaris* fruiting bodies (Yan et al., 2010; Chen et al., 2013; Dong et al., 2013). The carotenoid content of *C. militaris* is the highest known for the macrofungi (Yang and Dong, 2014). However, white mutants of *C. militaris*, with minimal carotenoid production have been reported (Shrestha et al., 2005; Jiang et al., 2011; Ma, 2014; Yang and Dong, 2014; Zhang et al., 2017). The carotenoid content of *C. militaris* albino strain was low; however, cordycepin and flavonoid contents and antioxidant activities were much higher than that of the normal yellow strains (Jiang et al., 2011; Zhang et al., 2017). There seems to be great potential for exploiting the albino *C. militaris* as a nutraceutical or functional food.

White edible fungi have become increasingly popular in the market because of their novelty and unique flavors. White Enoki (*Flammulina velutipes*) is the major commercial variety produced industrially in East Asia; nevertheless, wild Enoki mushrooms are yellow or deep brown colored as well. A white mutant strain of *Auricularia cornea*, named Yumuerin in Chinese, is another white variety cultivated extensively in China (Li and Zhang, 2016). Moreover, a white variety of *Hypsizygus marmoreus* is cultivated industrially on a large scale (Imtiaj et al., 2016).

The phenomenon and mechanism of albinism have been studied in some fungi. The orange pigmentation of the fungus *Neurospora crassa* is due to the accumulation of neurosporaxanthin and precursor carotenoids (Avalos et al., 2012). The photoinduction of carotenoids in *N. crassa* requires *de novo* synthesis of at least three enzymes, AL-1, AL-2, and AL-3, as characterized by the albino phenotypes of *N. crassa* mutants (Harding and Turner, 1981). Light induces the synthesis of carotenoids in *Fusarium fujikuroi*; however, the white collar protein, WcoA of *F. fujikuroi* is not essential for photocarotenogenesis (Estrada and Avalos, 2008).

The mechanism of albinism has been seldom studied in edible fungi. The color of the mycelium and fruiting body of *F. velutipes* is controlled by at least one major gene with multiple unlinked genes having additive effects. The development of color in *F. velutipes* is due to the accumulation of phenolic pigments in the fruiting body (Kitamoto et al., 1993). The molecular mechanisms of albinism in the edible fungi are worth studying because albino varieties are commanding an increasing share of the market. We have been focusing on the carotenoid biosynthesis pathway in *C. militaris*; however, homologs of two key enzymes (phytoene synthetase and phytoene dehydrogenase) in *N. crassa* were not found or cloned in the *C. militaris* genome (Yang et al., 2016), indicating differences in the biosynthetic pathways in these species.

*Cordyceps militaris* completes asexual and sexual reproduction with a few bioactive secondary metabolites when cultured *in vitro* (Shrestha et al., 2012). Furthermore, genome sequencing (Zheng et al., 2011), genetic transformation, and gene knockout (Yang et al., 2016) have been successful in this species, making it a model organism for the study of over 400 species of *Cordyceps* spp. that have been described (Shrestha et al., 2012). We previously reported that disruption of the gene encoding the photoreceptor CmWC-1 results in an albino mutation (Yang et al., 2016). An albino strain resulting from spontaneous mutation was also obtained during the fruiting body cultivation of *C. militaris*. In this study, the identity of the spontaneous albino mutant was ascertained by morphological observation and molecular phylogenetic analysis. The growth, morphology and metabolite production of the albino strain were also compared with that of the normal sibling strain. A comparative transcriptome analysis in response to light stress was performed between the two strains to reveal the genetic variation between the two strains.

## Materials and Methods

### Isolation and Identification of Albino and Sibling Strains

**FIGURE 1:**
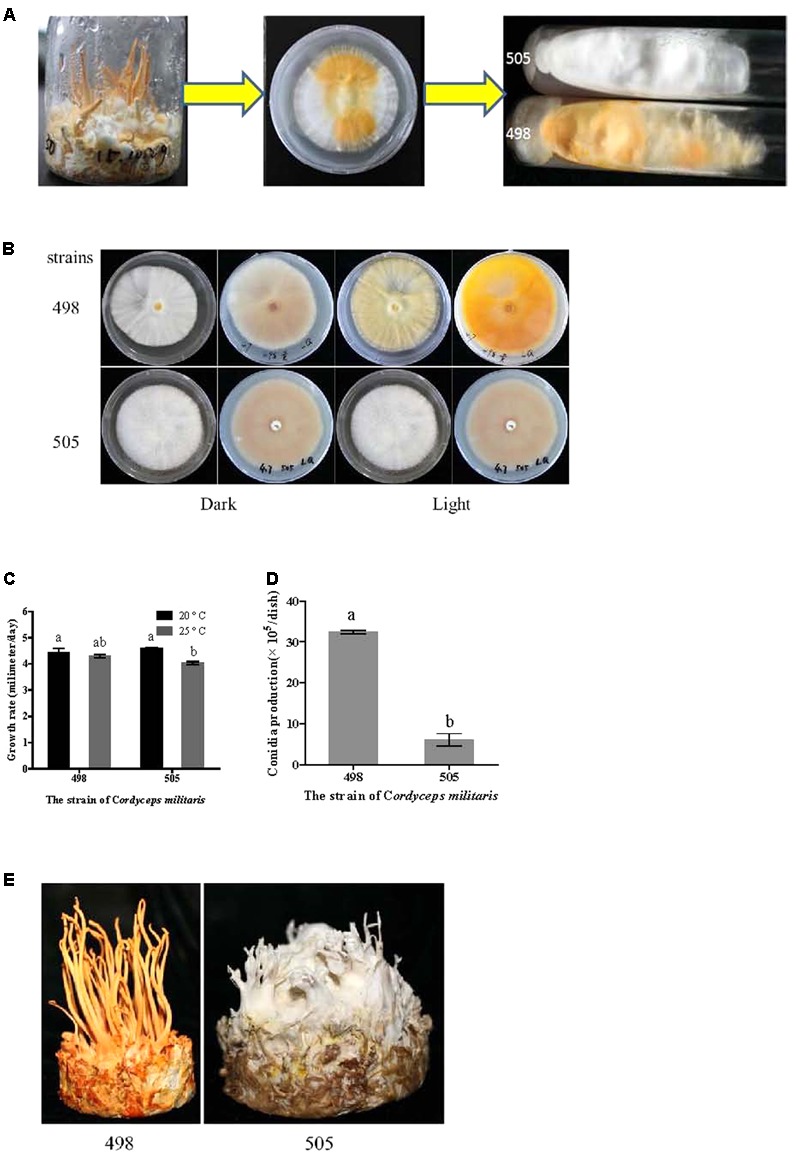
Isolation of albino and sibling strains and phenotype comparison. **(A)** Isolation of albino strain 505 and sibling strain 498. **(B)** Colony of strains 505 and 498 before and after light irradiation. **(C)** Growth rate of strains 505 and 498. **(D)** Condia production of strains 505 and 498. **(E)** Fruiting bodies cultivated on wheat medium. Different letters above the bars indicated significant differences (ANOVA followed by Duncan’s multiple range tests, *P* < 0.05).

During routine fruiting body cultivation of *C. militaris* CGMCC 3.16321 in the laboratory using standard methods (Zhan et al., 2006), we observed albinism (**Figure 1A**). Some tissue was excised from the albino fruiting body and incubated on potato dextrose agar (PDA) medium under natural light conditions at 20°C. After 10 days, two different colored tissues, yellow and white appeared on the plates (**Figure 1A**), from which the albino strain 505 (CGMCC 5.2191) and its sibling strain 498 (CGMCC 5.2190) were isolated (**Figure 1A**). These strains were sub-cultured 10 times to monitor trait stability.

For strain identification, genomic DNA was prepared with the cetyltrimethyl ammonium bromide (CTAB) method (Doyle and Doyle, 1987) and the internal transcribed spacer (ITS1-5.8S-ITS2) of the nuclear ribosomal DNA was amplified. The nucleotide sequences have been deposited in GenBank under accession numbers KY886359 and KY886361 for albino strain 505 and its sibling strain 498, respectively. Phylogenetic analysis based on the ITS sequence was performed to ascertain the identity of the strains using Mega 6.06 (Tamura et al., 2013).

### Comparison of Morphology, Growth Rate, and Metabolite Production Between the Albino and Sibling Strains

The mycelial growth of the two *C. militaris* strains was measured on PDA plates under dark conditions. Growth rate was calculated based on the colony diameter measured after 2 weeks of incubation. Six replicates were included for each strain. Conidia production was determined by scraping the mycelia of colonies from the PDA plates for 16 days and counting the conidia using a hemocytometer under a microscope (Nikon Eclipse 80i, Nikon Instruments Inc., Tokyo, Japan). Conidia production was calculated from six replicate plates with three counts for each strain. The cordycepin and carotenoid contents were determined following the previously optimized methods of Dong and Yao (2010) and Yang et al. (2014), respectively.

### RNA Extraction and Library Preparation

Strains 505 and 498 were incubated on PDA plates at 20°C for 14 days in the dark, and then half of the plates were cultured for another 3 days under white light (1750 Lux) until the mycelia of strain 498 turned orange. Fresh mycelia were collected for RNA isolation.

Total RNA was extracted using TRIzol reagent (Invitrogen, Carlsbad, CA, United States). RNA quality and concentration were evaluated using a Nanophotometer Spectrophotometer (Implen, CA, United States) and an RNA Nano 6000 assay kit for the Agilent Bioanalyzer 2100 system (Agilent Technologies, CA, United States). Twelve libraries (2 strains × 2 treatments × 3 biological replications) were generated using a NEB Next Ultra RNA Library Prep Kit for Illumina (NEB, Ipswich, MA, United States) following the manufacturer’s recommendations.

### Transcriptome Sequencing and Data Analysis

The cDNA libraries were sequenced on an Illumina HiSeqX-ten platform (Illumina Inc., United States) by Biomarker Technology Co., Ltd. (Shunyi, Beijing, China). Library quality was assessed on an Agilent Bioanalyzer 2100 system. Reads of 150 bp were sequenced from both ends using an Illumina HiSeq2000 instrument according to the manufacturer’s instructions.

Clean data were obtained by removing sequences containing adapters, poly-Ns, and low-quality reads from the raw data. The clean reads were mapped to the *C. militaris* genome (Zheng et al., 2011) using TopHat v2.0.9 (Trapnell et al., 2009). Differential expression analysis was performed using the Deseq2 tool (Love et al., 2014). The values in the matrix input were un-normalized counts of sequencing fragments. A corrected *P*-value of 0.05 and log2 (fold change) of 1 were set as the thresholds for significant differential expression, unless specified. A heat map was generated using the HemI software (Version 1.0.3.3, Heatmap Illustrator, Huazhong University of Science and Technology, Wuhan, China, Deng et al., 2014).

The GO enrichment analysis of the DEGs was performed using the GOseq R package based on Wallenius non-central hyper-geometric distribution, which can be adjusted for gene length bias in DEGs (Young et al., 2010). We also checked the significant enriched GO term manually. KOBAS software was used to test the statistical enrichment of DEGs in Kyoto Encyclopedia of Genes and Genomes (KEGG) pathways (Mao et al., 2005). The raw Illumina sequencing data were deposited in GenBank under Bioproject PRJNA390708.

### Quantitative Reverse-Transcription (RT)-PCR

RNA isolation and qRT-PCR were performed as previously reported (Yang et al., 2016; Wang et al., 2017). Relative gene expression levels were calculated using the 2^-ΔΔC_T_^ method (Livak and Schmittgen, 2001). The data obtained represent three biological replicates, and with two technical replicates each.

## Results

### Strain Identification and Phenotype Comparison

Albino strain 505 was isolated from a spontaneous mutation during fruiting body cultivation and the albino phenotype was stably maintained even after 10 subcultures. We named these strains 505 and 498, which had different colors but came from a common parent, the “albino” and “sibling” strains, respectively (**Figure 1A**). The ITS sequences of the two strains showed no variation and phylogenetic analysis based on the ITS sequence confirmed both strains were *C. militaris* (**Supplementary Figure S1**).

As shown in **Figure 1B**, the colony morphologies of the two strains grown in the dark were identical. In both the strains, the upper side of the plate was nearly white, while the reverse side of the plate showed a very shallow color under dark conditions. However, after light irradiation, the normal strain 498 appeared yellow on both the upper and reverse sides of the plate, but there was no color change observed for the albino strain 505.

The growth rates of the two strains at 20 and 25°C were determined based on the colony diameter (**Figure 1C**). Both the strains grew faster at 20°C than at 25°C; however, there was no significant difference between the two strains at the same temperature. Similar time courses of mycelial growth were observed at 20 and 25°C for the two strains (**Supplementary Figure S2A**).

In the normal strain 498, conidia were remarkably abundant under light conditions, approximately five times more than that observed in the albino strain 505 (**Figure 1D**). Both strains showed comparable conidial morphology and conidiophores (**Supplementary Figure S2B**).

Fruiting bodies were cultivated in both strains. Strain 498 grew well and produced yellow fruiting bodies, while the albino strain 505 only produced a few deformed fruiting bodies and appeared white during the entire growth period (**Figure 1E**). Fruiting body development seemed to be suppressed in the albino strain.

The contents of the two most important metabolites, carotenoids and cordycepin, in the two strains were determined. The carotenoid content in strain 498 was over 20 times higher than that observed in the albino strain 505 (**Table 1**). The opposite result was observed for the cordycepin content, which was significantly higher in the albino strain 505.

**Table 1:** Comparison between the contents of carotenoid and cordycepin in fruiting body of the albino and sibling strains.

Strain	Carotenoid content (μg/g)	Cordycepin content (mg/g)
505	103.96 ± 4.08	6.70 ± 1.41
498	2110.62 ± 250.14	3.09 ± 0.06

### Transcriptome Sequencing of *Cordyceps militaris* Albino and Sibling Strains

To compare the gene expression profiles of the albino and sibling strains in response to light stress, transcriptome sequencing was performed on strains 505 and 498 under both dark and light conditions. A total of 12 cDNA libraries were prepared from strains 498 and 505 after exposure to darkness (498D and 505D) and white light (498L and 505L) with three replicates each and subjected to Illumina deep sequencing.

A total of 296.3 million raw reads were generated by Illumina paired-end sequencing. After cleaning and quality checks, 277.6 million clean reads were obtained, with an average of 23.1 million reads per sample (**Table 2**). All Q30 percentages for the sequences (with an error probability of 0.01; a high-quality indicator) in the 12 libraries were over 85%. Almost 80% of the reads could be mapped to the *C. militaris* genome for each sample (Zheng et al., 2011).

**Table 2:** Summary of transcriptome sequencing.

Sample ID	Raw reads	Clean reads	Base number (bp) (G)	Q30 percentage (%)	GC percentage (%)	Mapped reads (% mapped)	Notes
498L-1	22627427	22182907	6.59	87.66	57.32	85.01	Replicate 1
498L-2	22667227	22051420	6.54	87.81	57.05	84.60	Replicate 2
498L-3	21266847	20760986	6.16	87.87	56.97	79.58	Replicate 3
498D-1	22646617	22109864	6.54	87.62	57.46	84.24	Replicate 1
498D-2	24431816	23580430	6.92	88.11	57.37.	82.12	Replicate 2
498D-3	27197107	26648306	7.90	87.16	57.66	85.77	Replicate 3
505L-1	24785024	23723197	7.04	88.43	57.00	84.33	Replicate 1
505L-2	26898104	21968052	6.50	87.50	57.49	83.74	Replicate 2
505L-3	29973805	22326194	6.62	86.84	57.22	83.13	Replicate 3
505D-1	26344323	25724533	7.63	87.14	57.57	85.89	Replicate 1
505D-2	25799958	25540010	7.63	86.25	57.54	87.86	Replicate 2
505D-3	21619244	21022281	6.22	87.33	57.74	84.77	Replicate 3

### Identification of Differentially Expressed Genes in the Transcriptomes

**FIGURE 2:**
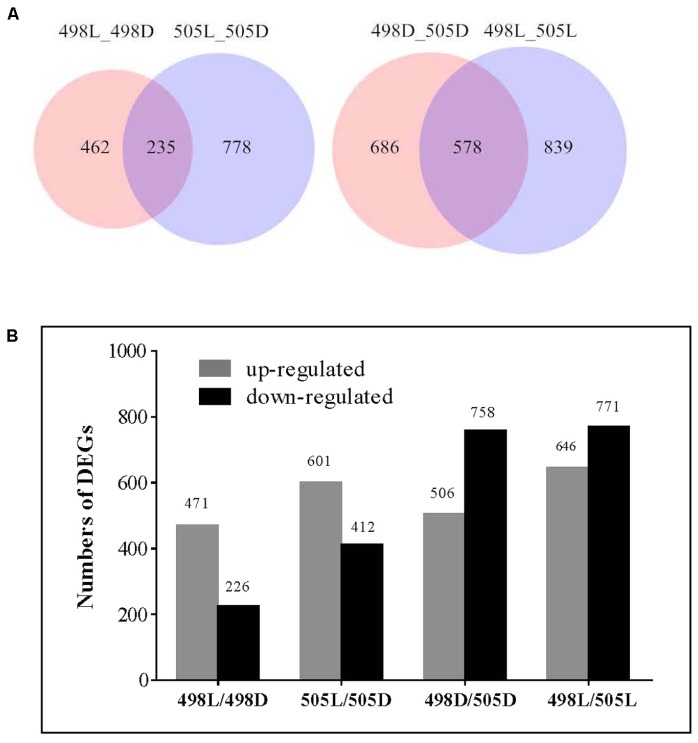
Differentially expressed genes in strains 498 and 505 under dark and light conditions. **(A)** Venn diagram illustrates the number of differentially expressed genes. **(B)** Differentially expressed genes in strains 498 and 505 under dark and light conditions. The number of genes differentially expressed is indicated on the top of the histograms.

Comparative transcriptome analysis was performed to identify differentially expressed genes (DEGs) between the tested samples. We observed that 697 and 1013 genes were significantly and differentially expressed for strains 498 and 505 in response to light (**Figure 2A** and **Supplementary Tables S1, S2**), representing 7.2 and 10.6% of the *C. militaris* genes, respectively. This indicated that additional genes were expressed in the albino strain in response to light, to reduce light impairment. Strains 498 and 505 shared 235 DEGs after the light treatment, indicating that these were important genes involved in the light response of *C. militaris.* Additionally, 462 genes were specific to strain 498, and these genes may be important in defining the phenotypic change in response to light stress. However, 159 (34.4%) of these were hypothetical protein genes, while 778 DEGS were unique to strain 505.

Compared with that of the normal sibling strain 498, 758, and 771 genes were up-regulated, and 506 and 646 genes were down-regulated under dark and white light conditions, respectively, in the albino strain 505 (**Figure 2B** and **Supplementary Tables S3, S4**). Among these, 578 DEGs were common under both conditions (**Figure 2A**), indicating that inherent differences in gene expression existed between two strains irrespective of light irradiation.

### Functional Distribution of Differentially Expressed Genes

**FIGURE 3:**
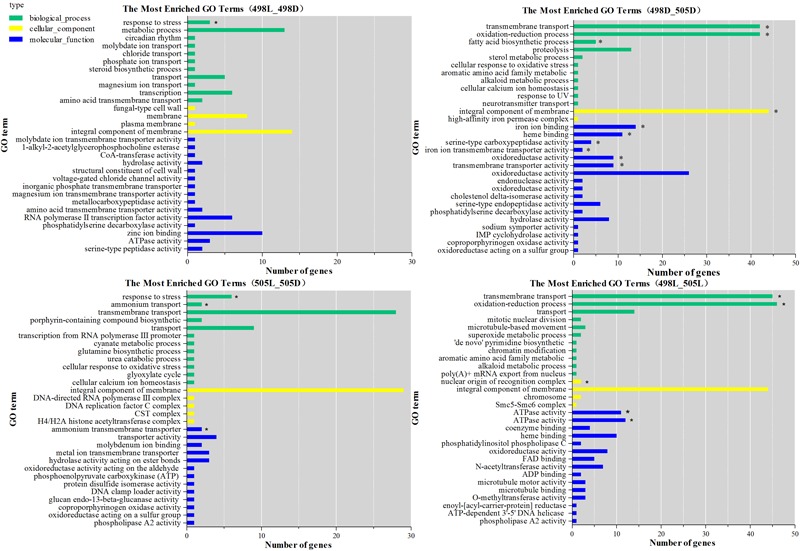
GO functional classification of differentially expressed genes. The green bars represent biological process; yellow bars represent cellular component; blue bars represent molecular function. The significance of *P*-value less than 0.005 was indicated by asterisk.

All DEGs were grouped into three functional categories: biological processes, cellular components, and molecular functions. There were 35 enriched GO terms for strain 505 in response to light stress (*P-*value 0.05), many more than in strain 498 (only 9 terms) (**Supplementary Table S5**). The most significantly enriched GO term in both strains in response to light stress was the biological process term “response to stress” (GO: 0006950, **Figure 3** and **Supplementary Table S5**).

When comparing strains 498 and 505, two biological process terms, “transmembrane transport” (GO: 0055085) and “oxidation-reduction process” (GO: 0055114), were significantly enriched under both, dark and light conditions. Under light conditions, when strain 498 was compared to 505, the top five functional categories were “transmembrane transport,” “ATPase activity, coupled to transmembrane movement of substances,” “oxidation-reduction process,” “ATPase activity,” and “nuclear origin of replication recognition complex” (**Figure 3** and **Supplementary Table S5**).

The DEGs from 498L vs. 498D, 505L vs. 505D, 498D vs. 505D, and 498L vs. 505L revealed 13, 21, 4, and 10 significantly enriched pathways, respectively (**Supplementary Table S6**). Most of the significantly enriched pathways (*P* < 0.05) from 498L vs. 498D were shared by 505L vs. 505D. However, 505L vs. 505D had some specific significantly enriched pathways (cmt00250 Alanine, aspartate and glutamate metabolism, cmt00310 Lysine degradation, cmt04139 Mitophagy – yeast, cmt 04146 Peroxisome, cmt 00440 Phosphonate and phosphinate metabolism), suggesting that the albino strain 505 had activated additional mechanisms in response to light stress.

The significantly enriched pathways of 498L vs. 505L were mainly associated with replication and repair, including cmt03420 nucleotide excision repair, cmt03430 mismatch repair, cmt03030 DNA replication and cmt03410 base excision repair (**Supplementary Table S6**). Most of the genes in these pathways were down-regulated in 498L compared to 505L, indicating that DNA repair mechanisms were generally more active in the albino strain 505 in response to light stress.

### Differential Expression of Photoreceptors in the Albino and Sibling Strains

Previously, we identified seven photoreceptors in the *C. militaris* genome (Yang et al., 2016). The expression trends of these photoreceptors were found to be similar in response to light in both strains 498 and 505 (**Table 3**). After light irradiation, the genes encoding VVD (CCM_04514), CRY-DASH (CCM_00774), and CPD photolyase (CCM_00151) were found to be up-regulated, which was consistent with our previous report (Yang et al., 2016). There was no differential expression observed between strains 498 and 505 either under dark or light conditions (corrected *P*-value of 0.001 and log2 fold change of 1).

**Table 3:** Differential expression of genes putative as photoreceptors in the genome of *Cordyceps militaris* between albino and sibling strains response to light stress.

Gene	Annotation	498L/498D		505L/505D		498D/505D		498L/505L	
		log2fold	padj	log2fold	padj	log2fold	padj	log2fold	padj
CCM_01180	CmWC-1	-0.078	0.917	-0.239	0.523	0.564	0.053	0.725	0.007
CCM_00072	CmWC-2	-0.908	0.154	-0.140	0.864	1.333	0.005	0.565	0.300
CCM_04514	VVD	7.850	0.000	9.932	0.000	1.595	0.004	-0.488	0.326
CCM_00774	CRY-DASH	2.299	0.000	2.072	0.000	-0.404	0.452	-0.176	0.756
CCM_02392	CRY-2	0.116	0.862	0.682	0.019	0.117	0.787	-0.449	0.134
CCM_00151	CPD photolyse	2.560	0.000	2.449	0.000	-0.051	0.954	0.061	0.935
CCM_04461	PHY	0.340	0.771	0.212	0.809	-0.088	0.926	0.040	0.962

### Differentially Expressed Genes Involved in the Secondary Metabolism in Response to Light in the Albino and Sibling Strains

A total of 28 secondary metabolite clusters were predicted in *C. militaris* using antiSMASH (Medema et al., 2011) including four terpene biosynthetic gene clusters. When the albino strain 505 was compared with strain 498, a gene encoding lanosterol synthase (CCM_09526) was up-regulated significantly, both under dark and white light conditions (a log2 fold change of 1 and corrected *P*-value of 0.001).

Potential secondary metabolite clusters were also identified using secondary metabolite unique regions finder (SMURF) (Khaldi et al., 2010). *C. militaris* possesses a number of secondary metabolite backbone-forming proteins, including nine polyketide synthases (PKSs), two PKS-like proteins, five non-ribosomal peptide synthases (NRPSs), eight NRPS-like proteins, three PKS-NRPS hybrid backbone synthases, and one dimethylallyl tryptophan synthase (DMAT).

**FIGURE 4:**
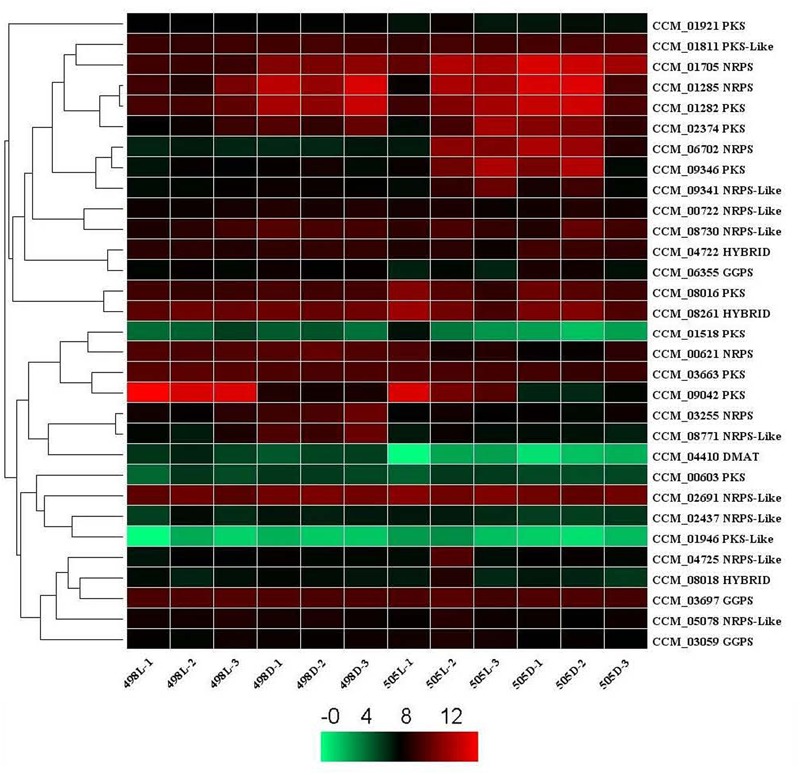
Expression of secondary metabolite backbone synthesis genes in *Cordyceps militaris*. Genes predicted to be involved in the synthesis of secondary metabolite backbones identified by SMURF. The levels of expression represent normalized values (log2). Genes with similar patterns of expression are clustered. NRPS, non-ribosomal peptide synthase; PKS, polyketide synthase; DMAT, dimethylallyl tryptophan synthase. HYBRID, hybrid PKS-NRPS enzyme. GGPS, geranylgeranyl diphosphate synthase.

When a corrected *P*-value of 0.001 and log2 (fold change) of 1 were set as the threshold for significantly differential expression, the gene encoding CCM_09042 (PKS) was up-regulated both in strains 505 and 498 in response to light stress. This gene was also among the top 10 DEGs for both strains after light irradiation, indicating its photo responsiveness. However, a gene encoding CCM_08771 (NRPS-like protein) that was down-regulated in strain 498 in response to light stress remained unchanged in strain 505 (**Figure 4** and **Supplementary Table S7**).

When the albino strain 505 was compared with strain 498, genes encoding DMAT (CCM_04410), an NRPS-like protein (CCM_08771), and 2 NRPSs (CCM_03255 and CCM_00621) were down-regulated, and NRPS (CCM_06702) was up-regulated under dark conditions (**Supplementary Table S7**). A gene for DMAT (CCM_04410) was down-regulated, but two NRPSs (CCM_06702 and CCM 01705) were up-regulated under light conditions (corrected *P*-value of 0.001 and log2 fold change of 1). CCM_04410 and CCM_06702 were common DEGs under both dark and light conditions, indicating that they were differentially expressed but were not affected by light stress.

### Differential Expression of Transcription Factor Genes in *Cordyceps militaris*

Putative transcription factor (TF) genes in *C. militaris* genome were identified using the annotation pipeline in FTFD, which annotates fungal TFs based on the InterPro database using DNA-binding motifs (Park et al., 2008). A total of 358 putative TF genes were identified, representing 3.7% of the 9651 protein-coding genes in *C. militaris* (**Supplementary Table S8**).

**FIGURE 5:**
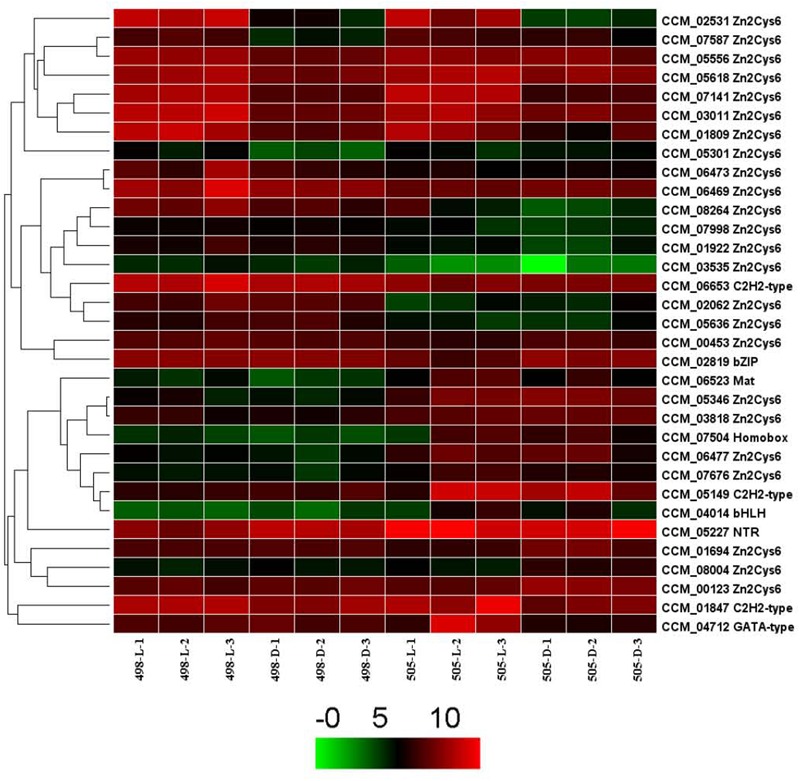
Expression of transcription factor genes in *Cordyceps militaris*.

We analyzed the expression patterns of these TF genes in strains 498 and 505 and found that the expression of 325 genes remained mostly stable in the two strains under different light conditions. The remaining 33 TF genes were chosen for further analysis (**Figure 5**). Among them, 24 TFs belonged to the Zn2Cys6 TF family. Four TFs, CCM_02531, 07141, 01809, and 03011 were up-regulated in both strains 498 and 505 in response to light, and all of them belong to the Zn2Cys6 TF family. CCM_05346, another Zn2Cys6 TF was up-regulated in strain 505 when compared to 498 under both dark and light conditions (**Figure 5** and **Supplementary Table S8**).

### Differentially Expressed of Post-modification Enzymes and Validation

Post-modification enzymes such as cytochrome P450s, aminotransferases, and methyltransferases are important for the formation of secondary metabolite compounds. By analyzing the genome, we identified 57 cytochrome P450s, 28 aminotransferases and 97 methyltransferases. Twelve of these genes with significantly differential expression between 498L and 505L are listed in the **Supplementary Table S9**.

**FIGURE 6:**
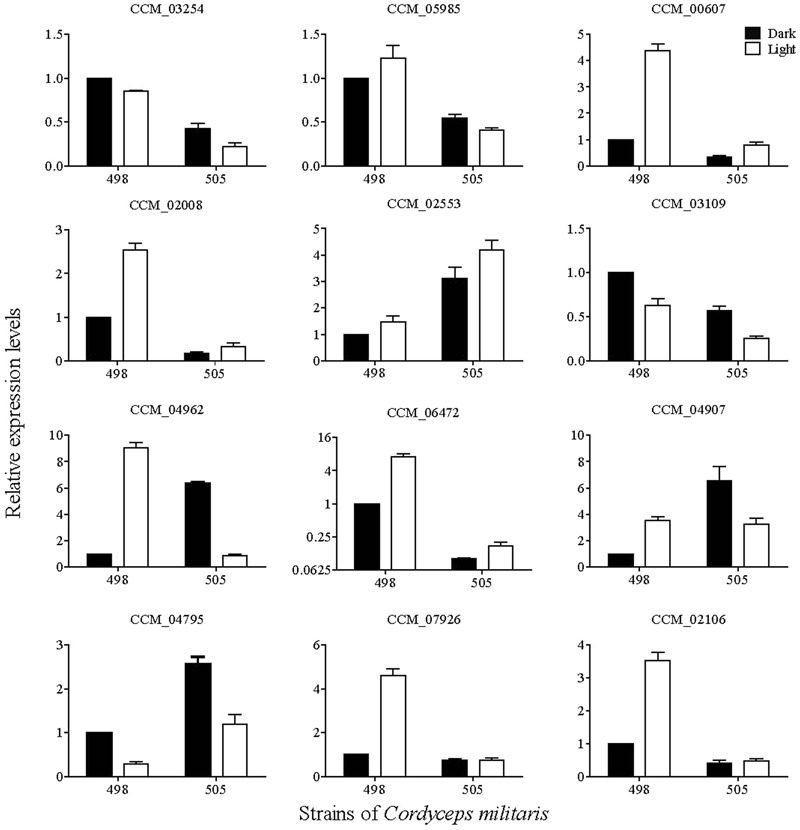
qRT-PCR analysis of key enzyme-coding genes involved in post-modification in *Cordyceps militaris*.

The expression levels of the 12 genes encoding post-modification enzymes were assessed by qRT-PCR (**Figure 6**). In general, the transcriptional changes of these genes according to qRT-PCR correlated with the transcriptome profiling data. Compared with 498L, all of the genes in 505L were differentially expressed to different degrees. Among them, CCM_06472 (*O*-methyltransferase) exhibited the highest expression level change between 498L and 505L (**Figure 6**). The expression of the gene encoding CCM_06472 (*O*-methyltransferase) increased significantly after light irradiation in strain 498 but not in the albino strain 505. The expression of CCM_06472 in strain 505 was almost 10 times lower than that in its sibling strain 498 both under dark and light conditions. Two Cyt P450s (CCM_00607 and CCM_02008) and two methyltransferases (CCM_02106 and 07926) showed light-induced expression in strain 498 but no significant change in the albino strain 505 after light irradiation was observed. The expression of the genes encoding CCM_02553 (Cyt P450) and CCM_04795 (methyltransferases) was found to be significantly increased in the strain 505 compared with that of strain 498, both under dark and light conditions. We postulated that these proteins may play important roles in the albinism of strain 505.

## Discussion

Albinism has been used for new variety screening and commercialization in some edible mushrooms, including white *F. velutipes, A. cornea*, and *H. marmoreus*. However, the molecular mechanisms of albinism in edible fungi have not yet been determined. During *C. militaris* fruiting body cultivation, a spontaneous albino mutant and its sibling strain with a normal orange color were obtained from a common parent. Morphology, growth rate, and metabolite production of the albino strain were compared with its sibling strain. Comparative transcriptome analysis was performed between these two strains in response to light stress. The results showed that many more genes were expressed in the albino strain to reduce light impairment, and that the significantly enriched pathways in 505L vs. 498L were mainly involved in replication and repair. The expression levels of some secondary metabolite backbone genes were found to be differentially expressed by over twofold in the albino compared with that of the normal strains. Zn2Cys6 TF and post-modification enzymes may be related to albinism of this strain. This report compared genome-wide transcriptional responses to light irradiation between an albino mutant and its sibling strain of an edible fungus, and the findings open new avenues for further investigation of the pigment biosynthetic pathway.

The albino strain 505 was obtained by spontaneous mutation, and its identity as *C. militaris* was ascertained by ITS phylogenetic analysis. The mutation ratio is not low because several independent researchers have reported albinism (Shrestha et al., 2005; Jiang et al., 2011; Ma, 2014; Yang et al., 2014). Besides the color differences in response to light stress, there were also other differences such as the conidia and metabolite production. Fewer conidia were produced in the albino strain 505 than in its sibling strain 498. The albino strain produces abnormal fruiting bodies. The carotenoid content of albino strain 505 was significantly lower that of its sibling strain 498, but the content of cordycepin in the fruiting body, a key metabolite in *C. militaris* was higher than that of strain 498. These results were also verified in another *C. militaris* albino strain, CGMCC 5.2189, maintained in our laboratory. Obviously, gene expression was altered in the albino strains.

Comparative transcriptome analysis revealed that albino strain 505 had many more DEGs in response to light stress than its sibling strain 498. Only 235 genes were shared by both strains, whereas 462 and 778 genes were specific to 498 and 505, respectively. This suggests that the albino strain and its sibling strain have different responses to light and that many more genes were photo responsive in the absence of pigment. KEGG pathway analysis revealed that the significantly enriched pathways in 498L vs. 505L were mainly involved in replication and repair. Previous studies have also reported that carotenoids provide photoprotection and are important to sustain yeast growth and enhance survival under high environmental levels of ultraviolet rays (Moliné et al., 2009). Furthermore, 578 DEGs were shared under both conditions, indicating some differences in gene expression between the two strains independent of light irradiation.

Light is an important factor involved in regulating carotenoid production in a wide variety of organisms. Mutations in the photoreceptors regulating carotenoid biosynthesis can yield albino phenotypes. In our previous study, inactivation of the photoreceptor gene *Cmwc-1* resulted in albinism (Yang et al., 2016). All the seven photoreceptors in the *C. militaris* genome showed almost the same differential expression mode between strains 498 and 505 in response to light stress. However, in the *ΔCmwc-1* albino strain, genes encoding the photoreceptors VVD, CRY-DASH, and cyclobutane pyrimidine dimer (CPD) photolyase were not induced by light (Yang et al., 2016; Wang et al., 2017), which differed from the observations on the spontaneous albino strain used in this study.

The expression levels of some secondary metabolite backbone biosynthesis genes including those encoding DMAT (CCM 04410), two NRPS-like proteins (CCM_06772 and CCM_08771), three NPRS (CCM_01705, CCM_03255 and CCM_00621), and lanosterol synthase (CCM_09526) were differentially expressed and over twofold in the albino compared with that of the normal strains. These genes might be related to the phenotype differences observed between the two strains. DMAT (CCM 04410) and NRPS (CCM_06702) were the common DEGs under both dark and light conditions, indicating that these genes were differentially expressed but not affected by light stress.

Given that the color of *C. militaris* fruiting body is attributed to the production of carotenoids, we were specifically interested in the expression of terpene biosynthetic gene clusters. A gene encoding lanosterol synthase (CCM_09526) was up-regulated under both dark and light conditions in the albino strain 505 in comparison to its sibling strain 498. Lanosterol synthase catalyzes the cyclization of (S)-2,3 oxidosqualene to lanosterol, a reaction that forms the sterol nucleus (Waage-Baudet et al., 2005).

The biosynthesis of carotenoids in *N. crassa* requires *de novo* synthesis of at least three enzymes, AL-1, AL-2, and AL-3 (Ávalos et al., 2014). Based on the carotenoid biosynthesis pathway in *N. crassa* (Ávalos et al., 2014), a putative carotenoid biosynthesis pathway for *C. militaris* has been proposed (**Supplementary Figure S3**), however, homologous of AL-2 and AL-3, the two key enzymes in *N. crassa* are absent in the *C. militaris* genome (Yang et al., 2016). The expression of known related genes was analyzed (**Supplementary Table S10**), but there was no significant differential expression between 498 and 505 after light irradiation. The biosynthetic pathway of carotenoids in *C. militaris* is our ongoing project.

The expression of most TF genes under dark and light conditions remained largely stable in the two strains. The differentially expressed TFs mostly belonged to the Zn2Cys6 TF family, which was also consistent with our previous report (Yang et al., 2016). Only CCM 05346, a Zn2Cys6 TF, was down-regulated in strain 498 compared with strain 505 under both dark and light conditions, and will also be the focus of future studies.

Post-modification enzymes such as *N*-methyltransferase, cytochrome P450, and aminotransferase are essential for production of secondary metabolites (Li et al., 2017). The modifications such as activation or silencing of genes are important for regulating secondary metabolism (Brakhage, 2013). It was found that almost half of the proposed 19 enzymes in the taxol biosynthesis pathway were cytochrome P450s (Croteau et al., 2006). The expression of *N*-methyltransferase gene is important for caffeine biosynthesis (McCarthy and McCarthy, 2007). We also studied the expression of genes related to post-modification enzymes, such as cytochrome P450, aminotransferases, and *N*-methyltransferases. Analysis of transcriptomic and qRT-PCR data revealed that some genes encoding cytochrome P450s and methyltransferases were differentially expressed in 498L and 505L, suggesting that they might be related to albinism, perhaps by regulating the expression levels of genes encoding post-modification enzymes. However, post-modification networks are complex and consist of hundreds of reactions that directly and indirectly interact (Li et al., 2017). Further research to better understand the downstream post-modification processes of carotenoid biosynthesis is needed.

In summary, the albino mutant and its sibling normal strains of *C. militaris* were obtained during fruiting body cultivation. We identified numerous light stress-associated DEGs in the two strains, respectively, and also compared the two strains by profiling their gene expression. These data suggest that the albino strain could accept light signal, and many more genes are differentially expressed in the albino strain in response to light stress to reduce the light impairment than its sibling strain. Genes coding for some secondary metabolite backbone, Zn2Cys6 transcription factor, cytochrome P450s, and methyltransferases may be responsible for the phenotypic variations observed. Findings from the current study offer a comprehensive understanding of albinism in *C. militaris.* Future work will focus on carotenoid biosynthetic pathway and its regulation in this fungus.

## Author Contributions

CD designed the experiments. QL, FW, and KbL performed the experiments. QL, FW, JZ, GL, and KL analyzed the data. CD wrote the manuscript. All authors reviewed the manuscript.

## Conflict of Interest Statement

The authors declare that the research was conducted in the absence of any commercial or financial relationships that could be construed as a potential conflict of interest.

## Supplementary Material

FIGURE S1 Phylogenetic relationships of albino mutant strain (505, CGMCC 5.2190) and its sibling strain (498, CGMCC 5.2191) and the other related species inferred from Neighbor-joining analysis of ITS rDNA sequences. The numbers at each node represent the percentage of bootstrap support calculated from 1,000 replicates. The sequence of “CGMCC 5.2190 and 5.2191” are indicated with solid triangle was generated in this study, while others were retrieved from GenBank. The ITS sequences of *Aspergillus terreus* (GU256759.1) and *A. fumigatus* (KU612366.1) were used as out groups.

FIGURE S2 Comparison of morphology and growth curve of two strains **(A)** Growth curve of two strains at 20 and 25°C. **(B)** The morphology of mycelium, conidiophore and conidia of two strains.

FIGURE S3 The putative carotenoid biosynthetic pathway in *Cordyceps militaris*.

TABLE S1 KEGG enriched pathway analysis of differentially expressed genes.

TABLE S2 Differentially expressed genes for strain 498 in response to light.

TABLE S3 Differentially expressed genes for strain 505 in response to light.

TABLE S4 Differentially expressed genes between strains 498 and 505 under dark condition.

TABLE S5 Differentially expressed genes between strains 498 and 505 under light condition.

TABLE S6 GO functional classification of differentially expressed genes.

TABLE S7 Differential expression of terpene biosynthetic gene clusters and secondary metabolites backbone genes.

TABLE S8 Differential expression of transcription factors genes in *Cordyceps militaris*.

TABLE S9 Differential expression of genes involved in post-modification enzymes in *Cordyceps militaris*.

TABLE S10 Differential expression of genes of putative carotenoid biosynthesis pathway in *Cordyceps militaris.*
