# Supplementary figures and images for: Comparative Transcriptome Analysis Between a Spontaneous Albino Mutant and Its Sibling Strain of Cordyceps militaris in Response to Light Stress
Source: Front Microbiol. 2018 Jun 8;9:1237. doi: 10.3389/fmicb.2018.01237 (PMC6002663; doi:10.3389/fmicb.2018.01237)

Fig.S1

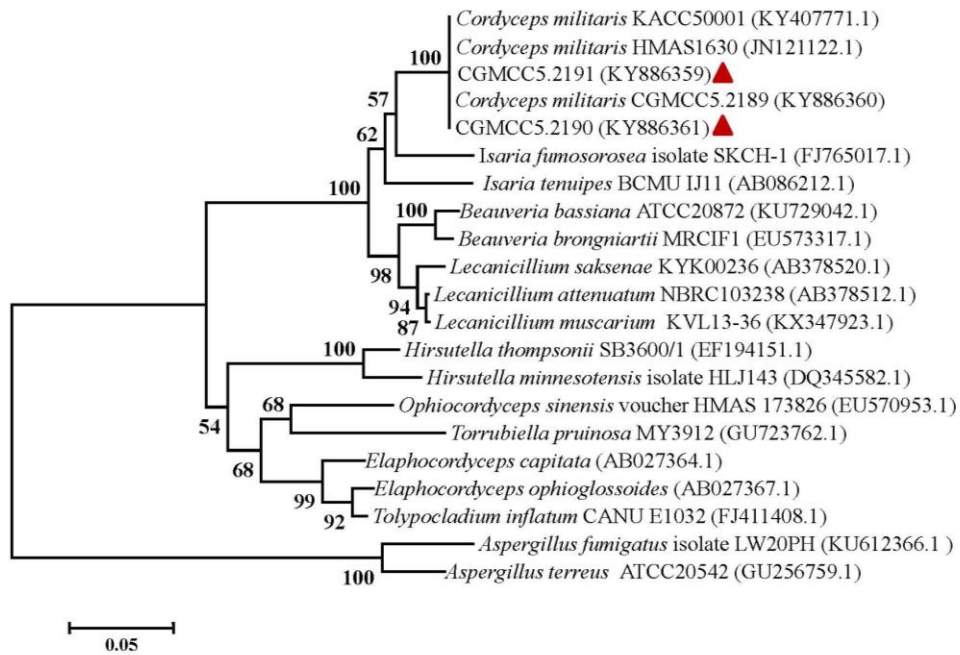

Fig. S2

A

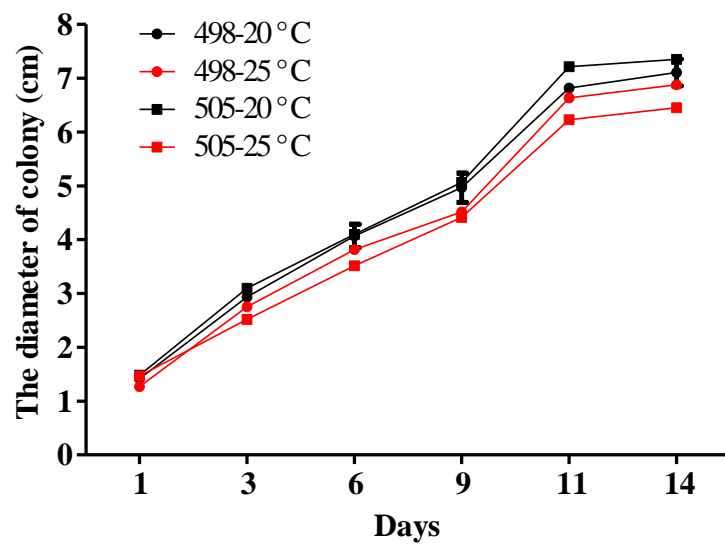

B

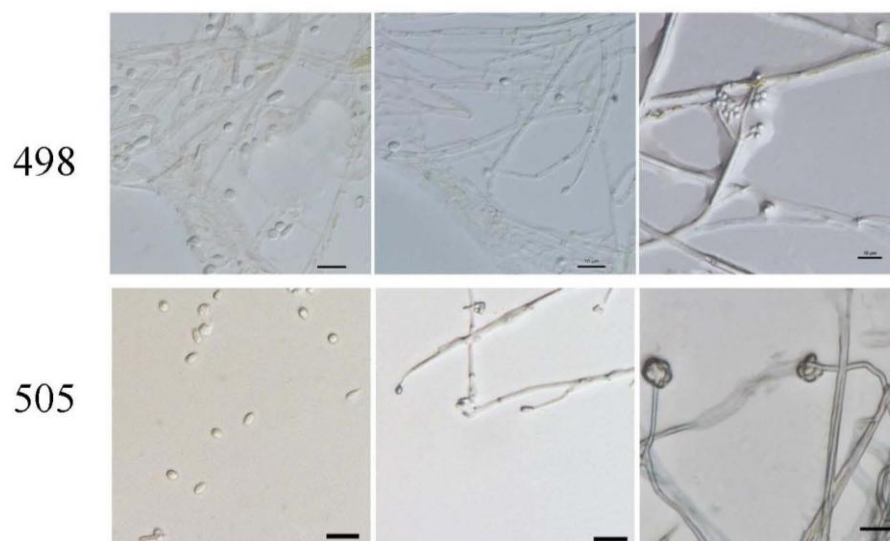

B

Fig. S3

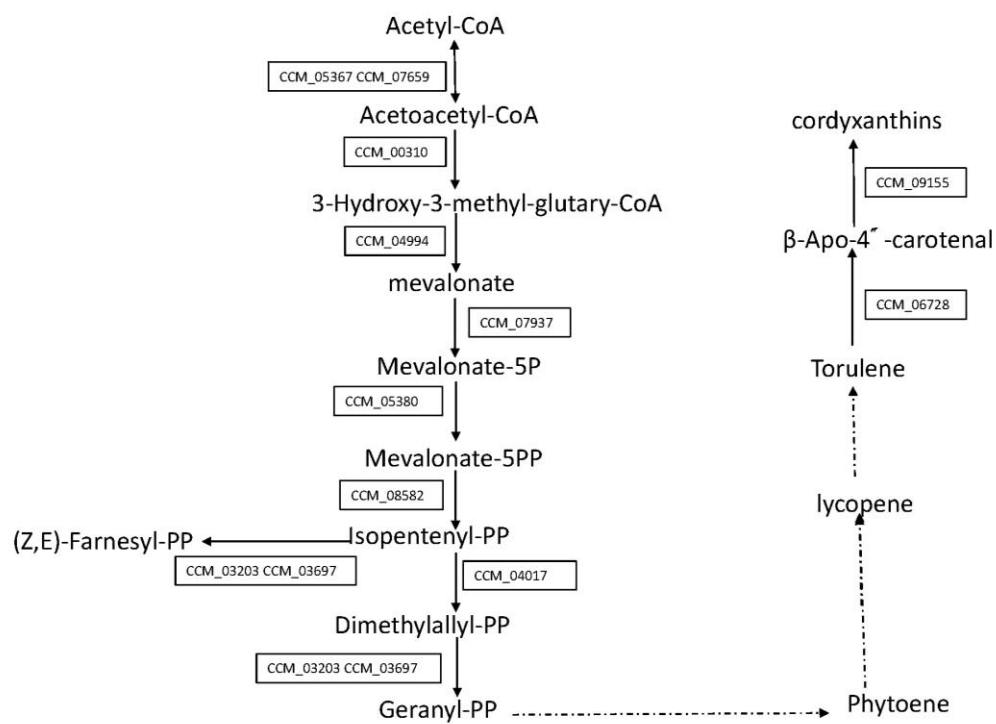

Supplement: FIGURE S1 — Phylogenetic relationships of albino mutant strain (505, CGMCC 5.2190) and its sibling strain (498, CGMCC 5.2191) and the other related species inferred from Neighbor-joining analysis of ITS rDNA sequences. The numbers at each node represent the percentage of bootstrap support calculated from 1,000 replicates. The sequence of “CGMCC 5.2190 and 5.2191” are indicated with solid triangle was generated in this study, while others were retrieved from GenBank. The ITS sequences of Aspergillus terreus (GU256759.1) and A. fumigatus (KU612366.1) were used as out groups. [file Image_1.PDF]
